# ELOVLs Predict Distinct Prognosis Value and Immunotherapy Efficacy In Patients With Hepatocellular Carcinoma

**DOI:** 10.3389/fonc.2022.884066

**Published:** 2022-07-15

**Authors:** Yu Zhang, Shujie Pang, Bo Sun, Minbo Zhang, Xiaoxiao Jiao, Linying Lai, Yiting Qian, Ning Yang, Wenzhuo Yang

**Affiliations:** ^1^ Department of Gastroenterology and Hepatology, Institute of Digestive Disease, Tongji Hospital, School of Medicine, Tongji University, Shanghai, China; ^2^ Department V of Hepatic Surgery, Eastern Hepatobiliary Surgery Hospital, Second Military Medical University, Shanghai, China

**Keywords:** hepatocellular carcinoma, ELOVLs, ELOVL1, prognosis, immunotherapy

## Abstract

**Background:**

Hepatocellular carcinoma (HCC) is a primary malignancy of the liver with high prevalence worldwide and poor prognosis. It has been verified that elongation of very-long-chain fatty acids gene family (ELOVLs), a group of genes that responsible for elongation of saturated and polyunsaturated fatty acids, participate in the pathogenesis and development of multiplex disease including cancers. However, the functions and prognosis of ELOVLs in HCC are still indistinguishable.

**Methods:**

First, we searched the mRNA expression and survival data of ELOVLs in patients with HCC *via* the data of The Cancer Genome Atlas (TCGA). The prognosis value of ELOVLs on HCC was assessed by Kaplan–Meier plotter and Cox regression analysis. reverse transcription quantitative- polymerase chain reaction (RT-qPCR), Western blot (WB), and immunohistochemistry were applied to assess the specific mRNA and protein expression of ELOVLs in HCC clinical specimens of our cohort. Then, the functional enrichment of ELOVL1 especially the pathways relating to the immune was conducted utilizing the Gene Ontology (GO), Kyoto Encyclopedia of Genes and Genomes (KEGG), and gene set enrichment analysis (GSEA) analysis. Additionally, TIMER, CIBERSOR, and tumor immune dysfunction and exclusion (TIDE) were employed to evaluate the relationship between ELOVL1 and immune responses. Last, the correlation of ELOVL1 with genome heterogeneity [microsatellite instability (MSI), tumor mutational burden (TMB), mutant-allele tumor heterogeneity (MATH), homologous recombination deficiency (HRD), purity, ploidy, loss of heterozygosity (LOH), and neoantigens] and mutational landscape were also evaluated basing on the date in TCGA.

**Results:**

Significant expression alteration was observed in ELOVLs family at the pan-cancer level. In liver cancer, ELOVL1 and ELOVL3 were strongly associated with poor prognosis of HCC by survival analysis and differential expression analysis. Immunohistochemistry microarray, WB, and RT-qPCR confirmed that ELOVL1 but not ELOVL3 played an important role in HCC. Mechanistically, functional network analysis revealed that ELOVL1 might be involved in the immune response. ELOVL1 could affect immune cell infiltration and immune checkpoint markers such as PD-1 and CTLA4 in HCC. Meanwhile, high expression of ELOVL1 would be insensitive to immunotherapy. Correlation analysis of immunotherapy markers showed that ELOVL1 has been associated with MSI, TMB, and oncogene mutations such as TP53.

**Conclusion:**

ELOVLs play distinct prognostic value in HCC. ELOVL1 could predict the poor prognosis and might be a potential indicator of immunotherapy efficacy in HCC patients.

## Introduction

Liver cancer is one of the most common cancers in the world and has a rising incidence worldwide. Hepatocellular carcinoma (HCC), accounting for almost 90% cases of all liver cancers, causes great global healthy problem (1, 2). Nowadays, the main treatments of HCC include surgical resection, chemotherapy, radiotherapy, and targeted therapy (3). Over the past decade, immunotherapy offers great promises in the treatment of a variety of malignancies including HCC (4). Preclinical and clinical investigations have revealed that various immunotherapies might extend current options for needed HCC treatment (5). However, the 5-year survival rate of HCC is still low for most patients fall to gain the optimal treatment due to no obvious clinical manifestations at an early stage (6). Thence, it is urgent to explore the specific molecular mechanisms underlying the pathogenesis of HCC and find diagnostic or prognostic biomarkers of HCC.

There are seven ELOVL enzymes (ELOVL1–7) in the ELOVLs family in mammals, which are mainly involved in catalyzing the extended cycle of the very-long-chain fatty acids (VLCFA) (7). ELOVLs have been verified to participate in the pathogenesis and development of diverse kinds of cancer. Although ELOVL1 was not upregulated in breast cancer comparing to paired normal breast, the gene silencing results demonstrated that ELOVL1 was essential for the growth of breast cancer cells (8). Additionally, study showed that the upregulated ELOVL1 promoted the accumulation of VLCFA in colorectal cancer tissues (9). ELOVL 2, a gene that is most highly closed to age prediction when appearing epigenetic alterations, contributes to aging by regulating lipid metabolism (10). Moreover, a previous study shows that ELOVL2 could attenuate tamoxifen resistance in breast cancer (11). ELOVL3 is responsible for the elongation of fatty acid in brown adipocytes and shows physiological roles in maintaining ocular homeostasis (12, 13). Mutations of dominant ELOVL4 are leading to the macular dystrophy of young mice, and ELOVL4 knockout mice would die soon after birth for the lack of skin barrier (14). The upregulation of ELOVL5 in mesenchymal-type gastric cancer cells causes to the sensitive to ferroptosis (15). Studies in liver-specific ELOVL6 knockout (LKO) mice have revealed that ELOVL6 decides hepatic insulin sensitivity and the length of ceramide acyl chain (16). ELOVL7 is confirmed to take part in the growth of prostate cancer (17). All these implied that abnormal ELOVLs family played an important role in disease by regulating lipid metabolism. However, the multiple expression landscape and pathological mechanisms of ELOVLs family had not been well investigated in HCC.

In the present study, we analyzed the expression and function of ELOVLs in HCC *via* bioinformatics and clinical tissues. The results identified the distinct expression patterns and prognostic values of ELOVLs family in HCC. Meanwhile, ELOVL1 showed correlation with immune envision and negative immune checkpoints PD-1 and CTLA-4. Furthermore, the high expression of ELOVL1 showed insensitive to immunotherapy and was related to microsatellite instability (MSI) and tumor mutational burden (TMB). Our work may offer novel comprehension on ELOVLs involved in HCC and uncover their underlying value in HCC treatment.

## Materials and methods

### Data Acquisition

The mRNA transcriptional data of HCC and normal liver tissues were obtained from The Cancer Genome Atlas (TCGA) (http://gdc.cancer.gov) datasets. All raw data were further analyzed after being standardized by log_2_ transformation. The R (version 4.0.3) and GraphPad Prism (version 7.0) software were utilized for analysis. We chose |log_2_ fold change (FC)|≥1 and adjusted p-value <0.05 as statistically significant genes.

### Patients and Clinical Database

A total of 113 HCC patients who underwent curative surgery in Eastern Hepatobiliary Surgery Hospital affiliated to Second Military Medical University from 2008 to 2014 were enrolled in this study. They were followed up postoperatively until December 2020. Patients confirmed of HCC by histopathological diagnosis and without adjuvant anticancer treatment such as radiotherapy and chemotherapy before surgery were chosen in the study. The clinicopathological data of selected patients were gained from the patient’s hospitalization records. The studies have obtained ethics approval by medical ethics committees of Tongji Hospital and Eastern Hepatobiliary Surgery Hospital, and all the patients involved in the study signed an informed consent form.

### Reverse-Transcription Quantitative PCR (qPCR)

Total RNA in liver of HCC patients and paired adjacent normal tissues was extracted by TRIzol reagent (Sigma-Aldrich, USA) under the instruction of the manufacturer’s protocol. The cDNAs were synthesized using a Prime Script TM RT kit (Takara, Japan) in reverse transcription reactions and the results were normalized to endogenous glyceraldehyde-3-phosphate dehydrogenase (GAPDH) expression. The primer sequences of ELOVL1 are the following: FORWARD (5′-3′) TTATTCTCCGAAAGAAAGACGGG, REVERSE (5′-3′) TTATTCTCCGAAAGAAAGACGGG. The primer sequences of ELOVL3 are the following: FORWARD (5′-3′) CTGTTCCAGCCCTATAACTTCG, REVERSE (5′-3′) GAATGAGGTTGCCCAATACTCC. The primer sequences of GAPDH are the following: FORWARD (5′-3′) AATGGGCAGCCGTTAGGAAA, REVERSE (5′-3′) GCGCCCAATACGA CCAAATC.

### Western Blot

Total protein was extracted with RIPA lysis buffer (Beyotime, China) mixed with a protease inhibitor (Beyotime, China) and centrifuged at 14,000*g* for 15 min at 4°C. Supernatant were quantified by the BCA Protein Assay Kit (Beyotime, China). Equal amounts of protein were separated, transferred onto polyvinylidene difluoride membranes, then blocked with 5% bovine serum albumin for 2 h at room temperature and incubated with primary antibodies against ELOVL1 (diluted 1:1,000, TA0670, Abmart, China) and GAPDH (diluted 1:25,000, 60004-1, Proteintech). The membrane was further reacted with horseradish peroxidase–conjugated secondary antibody (diluted 1:5,000, Beyotime, China) for 1 h at room temperature. The band intensity was analyzed by the ChemiDoc XRS systems (Bio-Rad Laboratories, United States) and Image J software.

### Immunohistochemistry

The tissue microarray was constructed using the paraffin-embedded tissue of the 113 patients. Tumor sections were incubated with primary anti-ELOVL1 (diluted 1:200, TA0670, Abmart, China) antibody in fridge overnight at 4°C. Then, the tissues were incubated with the secondary antibody (1:1,000 dilution, Thermo Fisher Scientific, A-10042, Massachusetts, USA) at 37°C for 1 h, and then covered by 3,3-diaminobenzidine (ZLI-9032, Zhongshan Biotech, China). Subsequently, all tissues were reviewed using the light microscope (Olympus 600 auto-biochemical analyzer, Japan). The positive cells score negative: 0%–5%; low: 6%–25%; medium: 26%–50%; high: >50%.

### Screening of ELOVL1 Expression and Functional Enrichment in HCC

To determine the differentially expressed gene (DEG) pattern between HCC patients with high and low ELOVL1expression, HCC patients in TCGA database were divided into high and low groups in line with the median ELOVL1 expression value. DEGs were determined by using the Limma package with the absolute value of logFC (logFoldchange) ≥1 and p-value ≤0.05. The functions of these identified DEGs were explored by hallmark gene sets, i.e., Gene Ontology (GO) gene sets and Kyoto Encyclopedia of Genes and Genomes (KEGG) pathway enrichment analysis. Gene set enrichment analysis (GSEA) was also analyzed using “clusterProfiler” package in R. The results were visualized using cluster Profiler and ggplot2 R packages (threshold: P < 0.05).

### Correlations Between ELOVL1 and Immune Environment

CIBERSOR was applied to explore the expression of ELOVL1 and relationship with the abundance of 22 tumor-infiltrating immune cells (TILCs) including CD8^+^ T cells, CD4^+^ T cells, B cells, neutrophils, macrophages, and dendritic cell by gene expression profiling in 44 cancer types from 10,180 samples (18). In addition, the TIMER database (https://cistrome.shinyapps.io/timer/) was also applied to inspect the correction between ELOVL1 and the abundance of TILCs through the “gene” module (19). The mRNA level of ELOVL1 and relationship with 60 immune checkpoints (24 inhibitory and 36 stimulatory) was identified by TISIDB database (http://cis.hku.hk/TISIDB/index.php) (20). The tumor immune dysfunction and exclusion (TIDE) was conducted to explore the correlation of ELOVL1 with TIDE scores and to predict the response possibility between ELOVL1-low and ELOVL1-high group to immune checkpoint inhibitor (21). Additionally, we analyzed the relationships of ELOVL1 expression with several clinical cohorts received immunotherapy through the TIDE website.

### Associations Between ELOVL1 Expression and Genome Heterogeneity (MSI, TMB, MATH, HRD, Purity, Ploidy, LOH, and Neoantigens) and Mutational Landscape

To identify the regulations of ELOVL1 expression and HCC, we first integrated ELOVL1 gene expression in TCGA pan-cancer (PanCAN, n = 10,535, g = 60,499). Second, as described in previous articles, TMB, MSI, homologous recombination deficiency (HRD), and neoantigen were used to evaluate the relationship between ELOVL1 and tumor mutation and treatment sensitivity (22). For TMB, we focused on the relationship between high expression of ELOVL1 and TMB. Last, mutant-allele tumor heterogeneity (MATH), purity, ploidy, and loss of heterozygosity (LOH) were used to inspect the association between ELOVL1 and tumor heterogeneity, as described in previous articles (23).

### Statistical Analysis

Survival curves were generated from the data in TCGA. The correlations between ELOVL1 expression and clinicopathological features were evaluated by Chi-square test evaluation. Kaplan–Meier survival curves were constructed using R (version 4.0.3). P < 0.05 was thought about statistically significant in the current study.

## Results

### Transcriptional Levels of ELOVLs Family in Patients With Cancer

**Figure 1:**
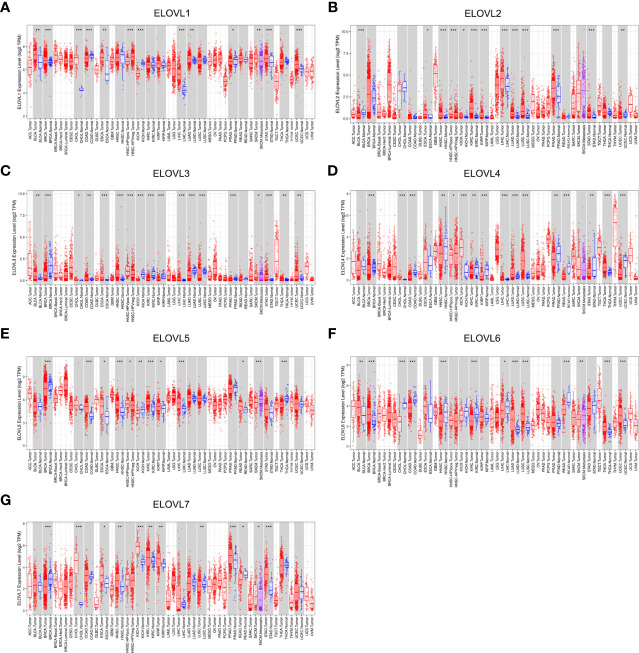
The mRNA expression of ELOVLs family in pan-cancer level. **(A–G)** ELOVLs family in various cancers was detected by TCGA database. *P < 0.05, **P< 0.01, and ***P < 0.001.

**Figure 2:**
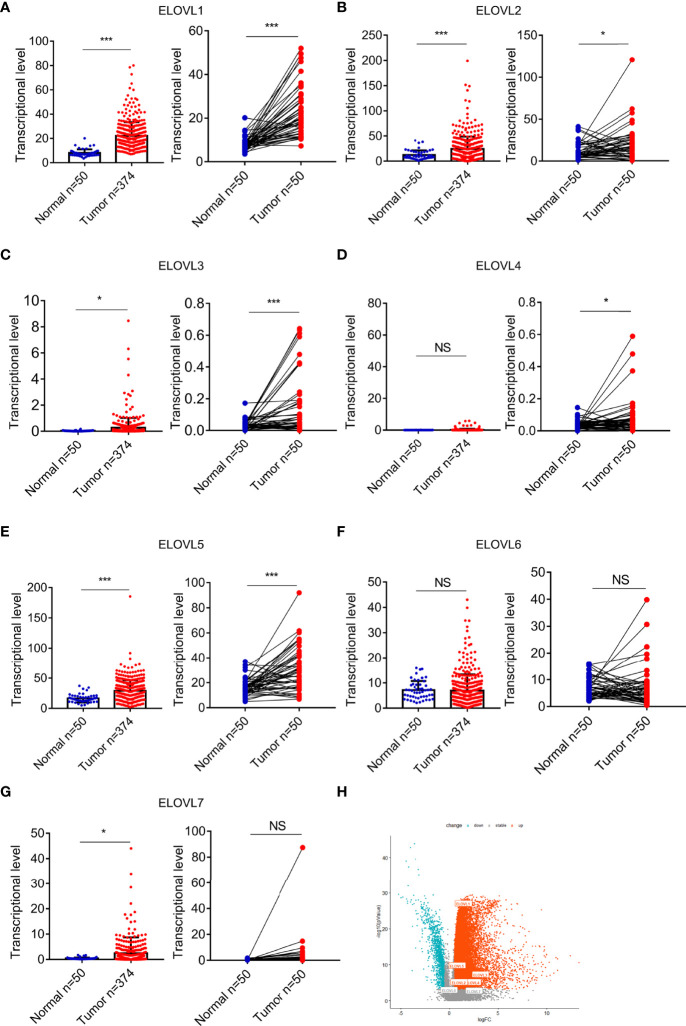
The mRNA expression of ELOVLs in HCC in the TCGA database. **(A–G)** ELOVLs were increased in HCC than the normal adjacent liver tissues except the ELOVL6. The left subfigures were the relative expression of the ELOVLs between the HCC and normal liver tissues from TCGA, and the right subfigures were the pairwise boxplot of the ELOVLs expression between the paired normal and HCC liver tissues in TCGA dataset. **(H)** The volcano plots of the expression of ELOVLs in HCC. *P < 0.05 and ***P < 0.001. NS, Not Significant.

At pan-cancer level, TCGA and GTEx database were utilized to explore the expression of ELOVLs between tumor and normal tissues. The results showed that the mRNA level of ELOVLs in most tumor tissues including HCC was higher than that in normal tissues (**Figures 1A–G**). Compared with normal tissues, ELOVL1, ELOVL2, ELOVL3, ELOVL5, and ELOVL7 were significantly increased in HCC (left subgroups, **Figures 2A–G**). In addition, ELOVL1, ELOVL2, ELOVL3, ELOVL4, and ELOVL5 were significantly overexpressed in the paired comparison between HCC and adjacent tissue (right subgroups, **Figures 2A–G**, **Figure 2H**). These data suggested that the ELOVLs family might play an important role in HCC.

### Increased mRNA Expression of ELOVL1 and ELOVL3 Predict Poor Prognosis in HCC

**Figure 3:**
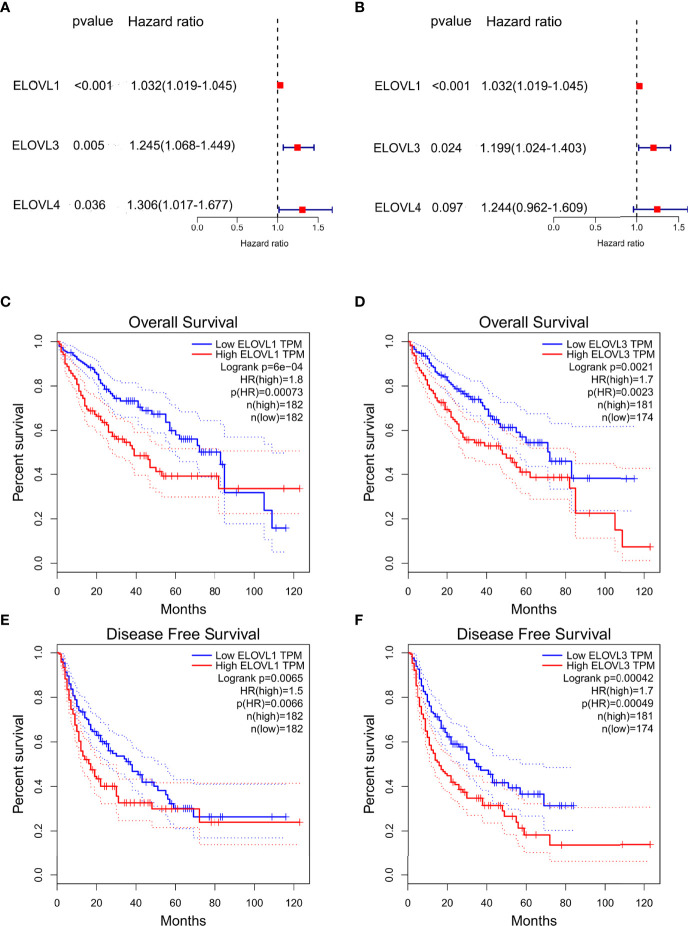
The prognostic value of ELOVLs family in HCC. **(A)** Univariate Cox regression analysis of ELOVLs in patients with HCC in TCGA database shows ELOVL1, -3, and -4 relating to the prognosis of HCC. **(B)** Multivariate Cox regression analysis of ELOVLs in patients with HCC in TCGA database shows ELOVL1 and ELOVL3 relating to the prognosis of HCC. **(C–F)** Overall survival (OS) and disease-free survival (DFS) of low- and high-expression groups of ELOVL1 and ELOVL3 in Kaplan–Meier Plotter.

Furthermore, univariate Cox regression (**Figure 3A**) and multivariate Cox regression (**Figure 3B**) analyses were used to analyze the role of ELOVLs family on the survival outcomes of HCC. Among the ELOVLs family, only ELVOL1 and ELOVL3 might have an impact on the survival of liver cancer patients. Consistent with this, the high expression of both ELVOL1 and ELOVL3 would reduce the overall survival (OS) (**Figures 3C, E**) and disease-free survival (DFS) (**Figures 3D, F**) of patients with HCC. These results suggested that ELVOL1 and ELOVL3 in the ELOVLs family were strongly associated with HCC and have the potential to predict the prognosis of patients with HCC.

### Overexpressed ELOVL1 in Liver Tissues From Patients With HCC Compared With Adjacent Normal Liver Tissues in 113 HCC Liver Tissues

**Figure 4:**
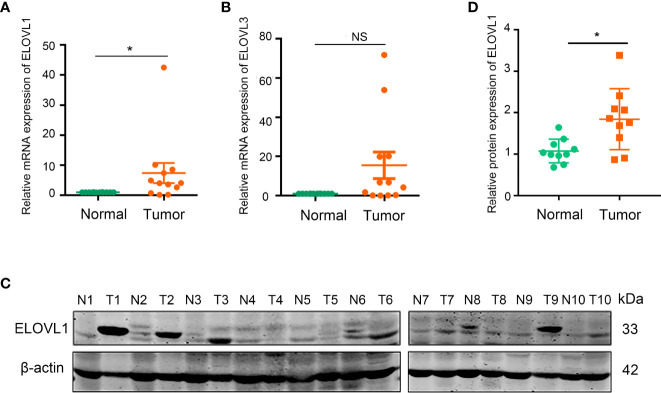
The mRNA and protein expression of ELOVL1 in HCC tissues and the adjacent tissues. **(A, B)** Comparing the adjacent tissues, the relative mRNA expression of ELOVL1 was increased and ELOVL3 was not changed in HCC tissues. **(C, D)** Protein expression of ELOVL1in HCC tissues comparing to adjacent tissues. *P < 0.05. NS, Not Significant.

To validate the results of the bioinformatics analysis in the ELOVLs family, we collected human samples of liver cancer and adjacent tissues. The results of RT-qPCR showed that the mRNA expression of ELOVL1, but not ELOVL3, was higher in HCC patients than of paired adjacent normal liver tissues (**Figures 4A, B**). Consistent with this, Western blot results showed that the protein expression of ELOVL1 was higher in HCC patients than of paired adjacent normal liver tissues (**Figures 4C, D**).

**Figure 5:**
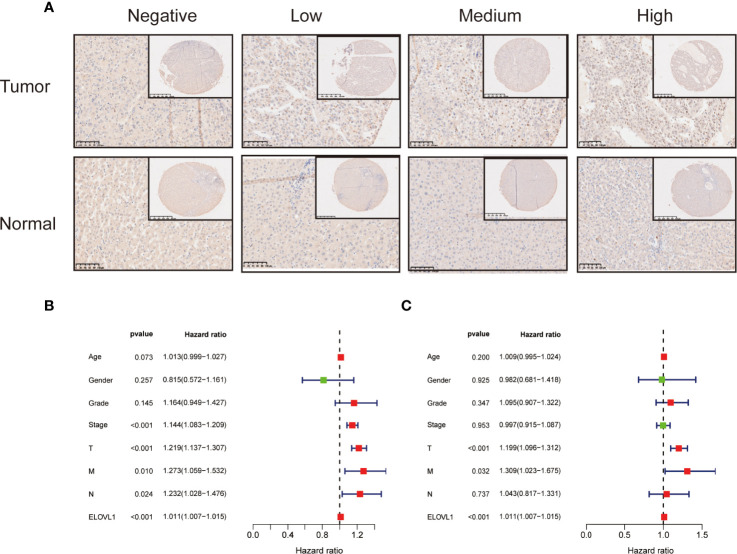
The immunohistochemical staining of ELOVL1 expression and Cox analysis of ELOVL1. **(A)** The immunohistochemical staining of ELOVL1 expression in HCC and normal adjacent liver tissues. **(B, C)** Univariate and multivariate Cox regression analysis of ELOVL1 and clinicopathologic variables of patients with HCC from TCGA database.

Furthermore, the immunohistochemical chip of 113 liver cancer samples showed 68 cases of them displayed high expression of ELOVL1 levels and 45 cases displayed low ELOVL1 expression levels (**Figures 5A**). Combined with bioinformatics analysis and human samples validation, these data indicated that ELOVL1 was significantly upregulated in HCC tissues compared with adjacent normal liver tissues, which suggested that ELOVL1 might play an important role in HCC.

### Association of ELOVL1 Expression With Clinicopathologic Characteristics and Prognosis of HCC Based on TCGA

Based on the expression profile of ELOVL1, we used transcriptome data from TCGA database to reclassify HCC patients into high-expressed ELOVL1 and low-expressed ELOVL1 groups. Then, logistic regression model was constructed to explore the relationship of ELOVL1 and clinicopathological characteristics in HCC patients. The results showed that ELOVL1was strongly associated with the tumor grade and tumor T stage of HCC patients (**Table 1**). Then, Cox proportional hazard models were constructed to explore the effects of ELOVL1 on patient survival times. The univariate Cox analysis showed that tumor stage (HR = 1.144, p < 0.001), T stage (HR = 1.219, p < 0.001), metastasis (HR = 1.273, p =0.010), N (regional lymph node) (HR = 1.232, p =0.024), and ELOVL1 expression (HR = 1.266, p < 0.001) were independent factors for OS of HCC patients (**Figure 5B**). Multivariate Cox regression analysis showed that ELOVL1 expression was confirmed to be a statistically significant predictor of OS in HCC patients (HR = 1.011, p < 0.001; **Figure 5C**). Highly expressed ELOVL1 in HCC patients would increase the survival risk and reduce the survival probability. In addition, T stage (HR = 1.199, p < 0.001) and metastasis (HR = 1.309, p =0.032) were also confirmed as an independent risk factor for OS.

**Table 1:** Correlation between ELOVL1 expression and clinicopathological characteristics of HCC patients in TCGA.

Clinicopathological Parameter	Total	Expression of ELOVL1		P-Value
		Low	High	
Gender				0.475
Male	108	63	45	
Female	236	148	88	
Age				0.825
<60	162	98	64	
≧60	182	113	69	
Tumor grade				0.001
G1-G2	213	146	67	
G3-G4	131	65	66	
Tumor stage				0.078
S1–S2	254	163	91	
S3–S4	90	48	42	
Tumor T stage				0.037
T1–T2	256	165	91	
T3–T4	88	46	42	
Lymph node metastasis				0.154
Yes	91	60	31	
No	253	151	102	
Metastasis				0.65
Yes	82	57	25	
No	262	154	108	

In conclusion, in terms of clinical applications, ELOVL1 was closely related to tumor grade and tumor T stage and might predict the prognosis of HCC patients.

### Functional Enrichment of ELOVL1

**Figure 6:**
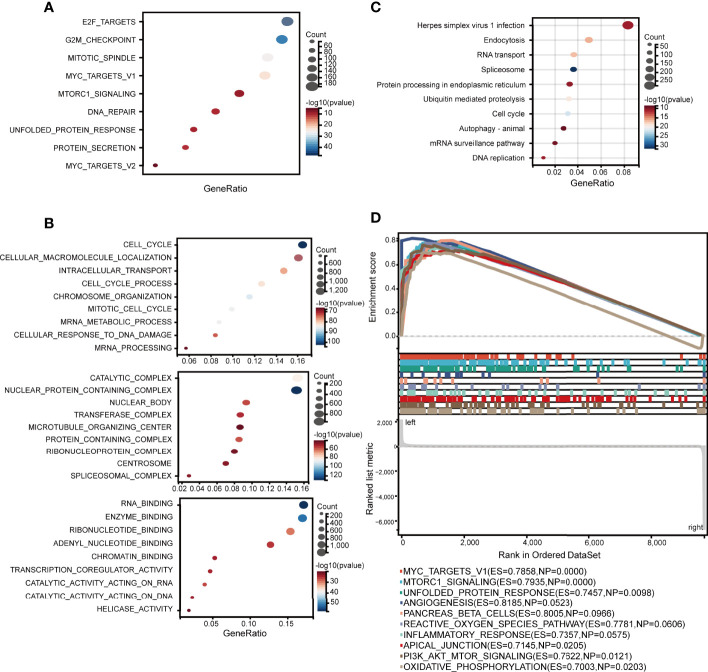
Associations between ELOVL1 and immune-related pathways. **(A)** Immune-related over-representation analysis. **(B)** Gene set enrichment analysis based on immune-related KEGG database; **(C)** Immune-related biological processes. **(D)** Immune-related molecular functions.

To identify how ELOVL1 participated in the tumorigenesis of HCC, we applied hallmark, GO, and GSEA analysis to explore the signaling pathways that involved in HCC. The hallmark enrichment analysis implied that ELOVL1 was involved in the E2F targets, G2M_checkpiont, mototic spindle, myc targets, MTORC1 signaling, DNA repair, unfolded DNA repair, and protein section. The GSEA study showed that ELOVL1 participated antigen processing and presentation (ES = 0.8987, NP = 0.0048), pathways in cancer (ES = 0.6627, NP=0.0124), leukocyte transendothelial migration (ES = 0.7697, NP = 0.0136), NOD-like receptor signaling pathway (ES = 0.8072, NP = 0.0187), and Toll-like receptor signaling pathway (ES = 0.7631, NP = 0.0260) (**Supplementary Figure 1**). Most of these pathways were related to inflammation and immune response, which is closely correlated with the tumorigenesis of HCC. Therefore, we further evaluated the relationship between ELOVL1 and the immune-related pathways. The results showed that ELOVL1 participated the antigen processing and presentation and innate immune response (**Figure 6A**). Besides, ELOVL1 also participated in major histocompatibility complex protein binding and adaptive immune response (**Figures 6B–D**). The results implied that ELOVL1 be involved in the tumorigenesis of HCC through immune microenvironment.

### Associations Between ELOVL1 and the Immune Microenvironment in HCC

**Figure 7:**
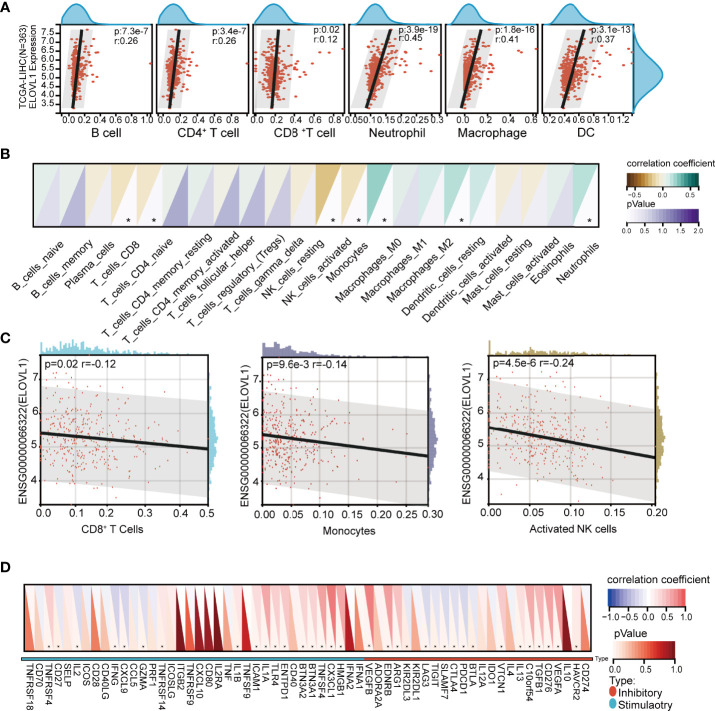
Correlation of ELOVL1 expression with immune cell infiltration and immune checkpoints in HCC. **(A)** Correlation of ELOVL1 expression with immune cell infiltration in HCC (TIMER). **(B, C)** Correlation of ELOVL1 expression with immune cell infiltration in HCC (CIBERSOR). **(D)** Expression of ELOVL1 and immunological checkpoints. *P < 0.05.

Based on the results of functional enrichment, we analyzed the immune components between high expression ELOVL1 and low expression ELOVL1 groups, which included immune cell infiltration and immune checkpoints. The results showed the mRNA expression of ELOVL1 was closely related to the immune cell infiltration, including CD8^+^ T cells, CD4^+^ T cells, neutrophils, macrophages, and dendritic cells through both the CIBERSOR and TIMER (**Figures 7A–C** and **Supplementary Figure 2**). In terms of immune checkpoints, the heat maps illustrated that ELOVL1 were closely connected with immune checkpoint. ELOVL1 was positively correlated with PD-1 (PDCD1), CTLA4, LAG3, endothelial growth factors (VEGFs), and so on, which are the common immune checkpoints in the HCC tumor-immune microenvironment (TIME) (**Figure 7D**).

**Figure 8:**
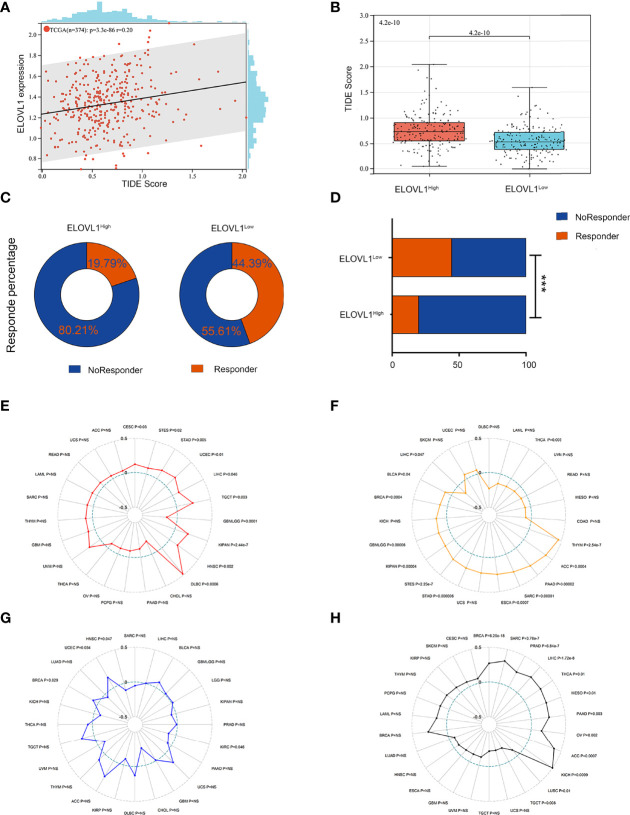
Relationship between ELOVL1 and response to immunotherapy. **(A)** The associations between ELOVL1 expression and TIDE score. **(B)** High ELOVL1 was positively correlated with high TIDE score. **(C, D)** High ELOVL1 group had a higher proportion of tumor immune dysfunction and rejection. **(E)** Relationship between ELOVL1 and MSI. **(F)** Relationship between ELOVL1 and TMB. **(G)** Relationship between ELOVL1 and neoantigen. **(H)** Relationship between ELOVL1 and HRD. ***P < 0.001.

Next, we performed the TIDE analysis of the effect of ELOVL1 on immunotherapy. These results showed that ELOVL1 had an obvious positive correlation with the TIDE score (**Figures 8A, B**). Specifically, patients with elevated ELOVL1 levels were more likely to show no response to immunotherapy according to the results from the TIDE analysis, whereas patients with low ELOVL1 level may be more sensitive to immunotherapy treatment (**Figure 8C–D**). All these results implied that ELOVL1 might participate in HCC *via* immune evasion and might predict the efficacy of immunotherapy.

.

### Correlations Between ELOVL 1 Expression and Immune Biomarkers in HCC

Studies have shown that indicators of genome heterogeneity might be the biomarker of immunotherapy, so we analyzed the correlations between ELOVL 1 and genome heterogeneity such as MSI, TMB, and neoantigen in HCC (24–26). As shown in **Figures 8E–H**, ELOVL1 is positively related to MSI and HRD (R = 0.298967888875077, P = 1.71923786304186e-8) in eight cancers including HCC (R = 0.104455471439159, P = 0.0455298564418963). Meanwhile, ELOVL1 is negatively related to TMB (R = −0.12, P = 0.047). However, ELOVL1 was not connected with neoantigen. MSI and TMB are the most studied tumor immunotherapy markers in cancers including HCC. In the correlation between ELOVL1 and MSI, TMB illustrated that ELOVL1 might be the biomarker of the efficacy of immunotherapy, which is consistent with the former results. As for the tumor heterogeneity, ELOVL1 was positively related to MATH (R = 0.15, P = 0.020), tumor ploidy (R = 0.12, P = 0.029), and LOH (R = 0.38, P = 0.001) in HCC. Additionally, ELOVL1 was negatively related to tumor purity (R = −0.11, P = 0.035) in HCC (**Supplementary Figure 3**). A few studies implied that oncogenic pathways driven by genetic mutations were also related to immune microenvironment, which, in turn, could impact response to immunotherapies in several types of cancer including HCC (27, 28). Thus, the mutation database was used to analysis the associations between ELOVL1 and mutational landscape in HCC. As shown in **Supplementary Figure 4**, ELOVL1 was related to ADRA1D, POTEH, GRIN1, NLRP12, SHANK1, MYH7, ZFPM2, FAM47A, TSC2, RB1, CSMD1, CTN, NB1, and TP53, which were common mutations in HCC. The relationship of MSI, TMB, and mutational landscape indicated that ELVOL1 had the potential to predict the efficacy of immunotherapy of HCC.

## Discussion

HCC is an aggressive disease with poor survival outcomes for patients with advanced/metastatic condition just receiving standard treatments (6). Hepatic immune system relating tumor microenvironment exhibits great impact on preventing progressing and treatment resistance of HCC (29, 30). Immunotherapy has the capacity to avoid immune tolerance mechanisms and strengthen antitumor ability comparing with standard treatments (4, 31, 32). The recommendation of immunotherapy strategies including immune checkpoint inhibitors, whether as single agents or combining approved local and systemic treatments, has notably altered the therapeutic outcome of HCC in recent years (33). The combination of the immune checkpoint inhibition of programmed death-ligand 1 (PD-L1) atezolizumab and the VEGF neutralizing antibody bevacizumab has become a first-line therapy for patients with advanced HCC (34). However, some patients show no respond to currently available immunotherapy (5, 35). Therefore, it is urgent to find biomarkers to assess the efficacy of immunotherapy and guide the precise clinical treatment of patients with HCC. ELOVLs have been identified basing on their substrate specificity and protein motif sequences (36). Although a few of studies have threw some light on the relationships between ELOVLs and lipid metabolism in non-alcoholic fatty liver disease (NAFLD) and cancers, the impact of ELOVLs in HCC still remains unclear. Aiming to reveal the underlying functions and distinct prognosis value of ELOVLs in HCC, we explored the public data set *via* bioinformatics analysis to offer novel insights for future research in the present study.

In the present study, we found that ELOVLs were upregulated in HCC except ELOVL6 and ELOVL7 *via* data from TCGA. The univariate and multivariate Cox regression analyses revealed that high ELOVL1 and ELOVL3 could be independent factors for OS and DFS in HCC especially in the first 5 years. The upregulated expression of ELOVL1 and ELOVL3 predicted poor prognosis in HCC. However, the verification *via* the liver tissues of patients with HCC in our cohort showed that only ELOVL1 was upregulated. In addition, high ELOVL1 expression was remarkably associated with advanced TNM stage and tumor grade. These outcomes suggest that ELOVL1 could be a potential biomarker in diagnosis and prognosis of HCC patients. The functional network revealed that the signaling pathway involved in ELOVL1 was related to inflammation and immune response. As a typical inflammation-linked tumorigenesis, immune evasion is one of the features occurring during the initiation and evolution of HCC (37). The immune evasion of HCC was mediated by different mechanisms such as fostering an immunosuppressive microenvironment or mediating cytotoxic cell dysfunction (38). CD8^+^ T lymphocytes are the primary cytotoxic tumor-infiltrating lymphocyte subset in HCC, and the immune checkpoints PD-1, CTLA-4, and LAG-3 are negative regulators of CTL function (39, 40). In the present study, the immune cell infiltration analysis illustrated that ELOVL1 was related to most kinds of immune cells related to immune envision. Specially, the results of relationship with CD8^+^ T cells are different by CIBERSOR and TIMER, which is a algorithmic statistical problem and needs further verification in clinical specimens and *in vitro* cells (19). The positive correlations between ELOVL1 and inhibitory immune checkpoints and high TIDE score analysis indicated that ELOVL1 might participate in the immune evasion of HCC. Furthermore, the high level of ELOVL1 was more likely to show no response to immunotherapy and association with MSI and TMB implied that ELOVL1 could predict the efficacy of immunotherapy. At the same time, high expression of ELOVL1 was related to the mutation of TP53 (p53), which played dual role in immune regulation and might be applied to optimize immune checkpoint inhibitor therapy for cancer treatment (41). All these revealed that ELOVL1 might play a pivotal role in immunosuppression in HCC development and could be a potentially novel biomarker to predict the efficacy of immunotherapy against HCC.

As an indispensable component of immunotherapy, the TIME has gradually acquired accumulative attention, and the analysis of TIME will contribute to the improvement of immunotherapy responsiveness (42). Some researchers revealed that the TIME could be taken as a main prognostic indicator, which could also enhance the potential of precision treatments (43). Although immunotherapy achieved great advances in HCC treatment, precise markers for patients to benefit from anti-PD1 or anti-CTLA4 therapy were still absent. Recent work had thrown some light on this issue. Dai et al. used 11 differentially expressed immune-related genes in 361 HCC patients to construct immune-related gene-based prognostic index, which can predict the survival of HCC patients and the response of immunotherapy (44). The study of Zou et al. illustrated that CDK1, CCNB1, and CCNB2 are potential prognostic biomarkers of HCC. CDK1, CCNB1, and CCNB2 may potentially be able to predict the response to immunotherapy, and combining immunotherapy with inhibitors of these genes may improve the curative effect (45). Moreover, a study constructed a score based on alpha-fetoprotein and C-reactive protein to predict disease control rate and OS in immune checkpoints inhibitors (ICI)-treated patients with HCC (46). In the present study, we found that ELOVLs acted as a dismal prognosis marker and ELOVL1 might be a potential biomarker of immunotherapy efficacy of patients with HCC. The expression and functions of ELOVLs especially ELOVL1 could be investigated in more retrospective clinical design and even the prospective clinical cohorts. The expression of ELOVL1 could benefit patients for immunotherapy and support decision-making in daily clinical practice. In addition, we would focus on how ELOVL1 influences the efficacy of ICIs in HCC treatment in our further work.

There are still a few limitations in the present study. First, the clinical data were limited and retrospective, and the specific expression and function of ELOVLs in HCC should be verified in prospective design. In addition, ELOVL6 was inconsistent with the results of other researchers.They found that ELOVL6 enhanced oncogenic activity in liver cancer and indicated poor prognosis in patients with HCC, whereas the results of ours showed no significance (47). The relationship between ELOVL6 and HCC in the present study was *in silico* and needs further verification. We need enough clinical data to correct our results in future studies.

## Conclusion

In conclusion, ELOVLs play distinct prognostic value in HCC and ELOVL1 serves as a dismal prognosis biomarker in HCC patients. In addition, ELOVL1 participates in the development and progression of HCC mainly *via* pathway-related immune response. Moreover, ELOVL1 was associated with immune cell infiltration, immune checkpoints, and response to immunotherapy. It is likely to be a novel therapeutic target in combination with immunotherapy and potential target to predict the efficacy of immunotherapy of HCC.

## Data Availability Statement

The authors acknowledge that the data presented in this study must be deposited and made publicly available in an acceptable repository, prior to publication. Frontiers cannot accept a article that does not adhere to our open data policies.

## Ethics Statement

The studies involving human participants were reviewed and approved by Ethics Committee of Eastern Hepatobiliary Surgery Hospital. The patients/participants provided their written informed consent to participate in this study.

## Author Contributions

YZ, SP, and BS planned, designed and performed the experiments and wrote the manuscript. MZ and XJ analysed the data and involved in manuscript preparation. LL and YQ drafted the figures and table. NY and WY supervised the research and checked the manuscript. All authors contributed to the article and approved the submitted version.

## Funding

This study was supported by funds from the National Natural Science Foundation of China (grant numbers: #81873567 to WY), Natural Science Foundation of Tongji Hospital Affilatied to Tongji University (#TJ2004 to WY) and The State Key Project for Liver Cancer (2012ZX10002017-004,2017ZX10203205-001-002).

## Conflict of Interest

The authors declare that the research was conducted in the absence of any commercial or financial relationships that could be construed as a potential conflict of interest.

## Publisher’s Note

All claims expressed in this article are solely those of the authors and do not necessarily represent those of their affiliated organizations, or those of the publisher, the editors and the reviewers. Any product that may be evaluated in this article, or claim that may be made by its manufacturer, is not guaranteed or endorsed by the publisher.
