# Supplementary figures and images for: ELOVLs Predict Distinct Prognosis Value and Immunotherapy Efficacy In Patients With Hepatocellular Carcinoma
Source: Front Oncol. 2022 Jul 15;12:884066. doi: 10.3389/fonc.2022.884066 (PMC9334671; doi:10.3389/fonc.2022.884066)

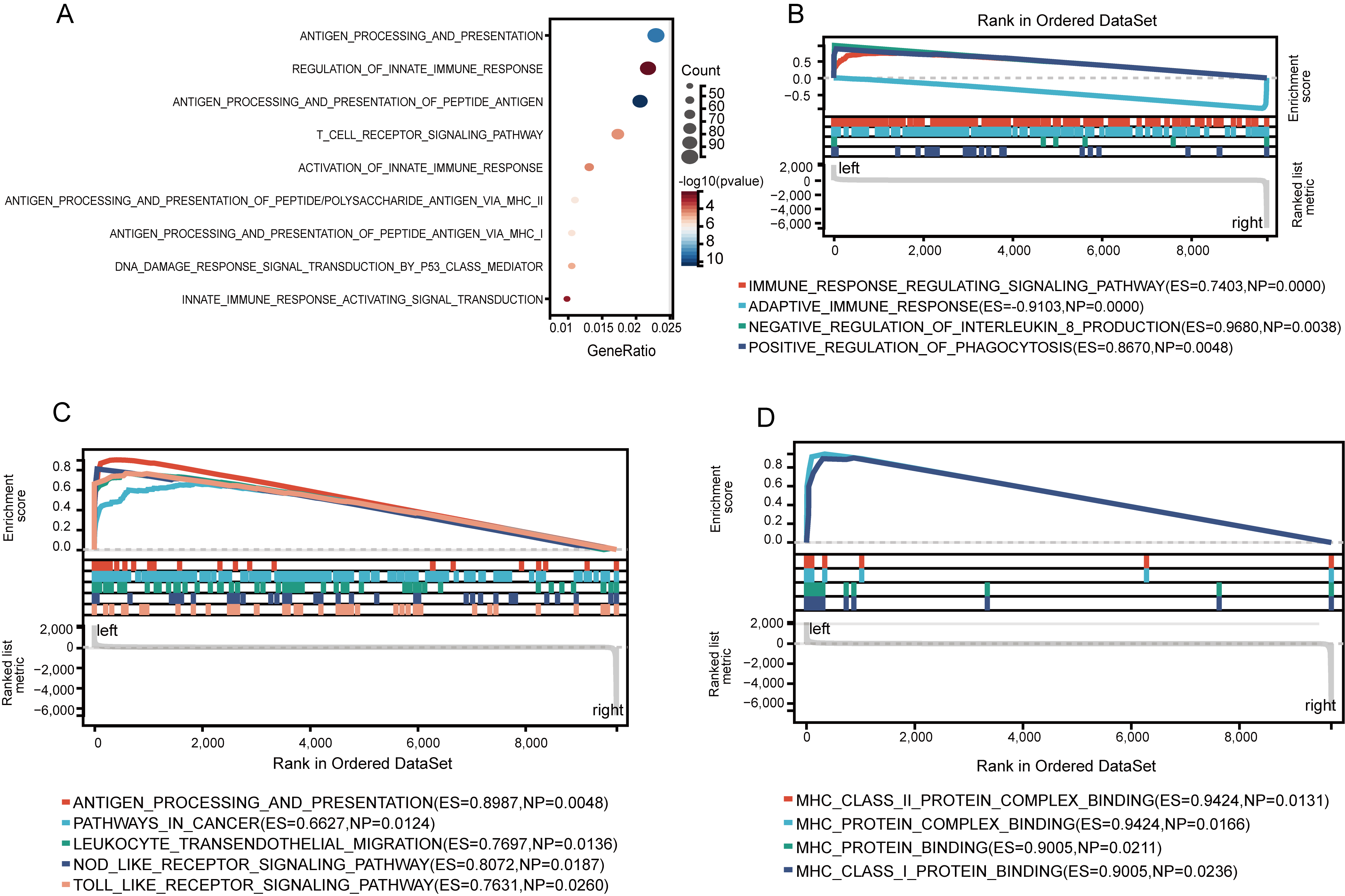

Supplement: Supplementary file 1 [file Image_1.tif]

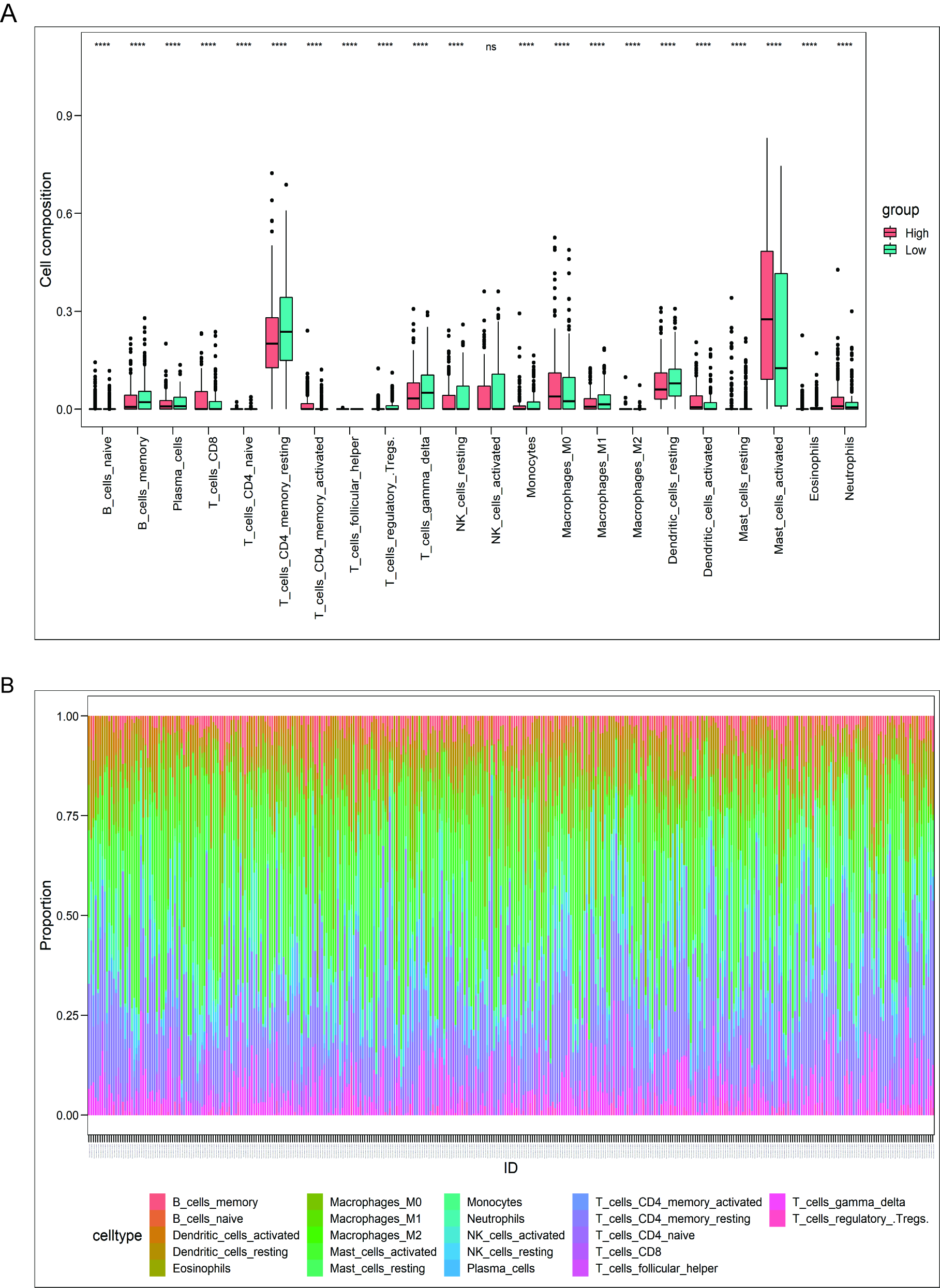

Supplement: Supplementary file 2 [file Image_2.tif]

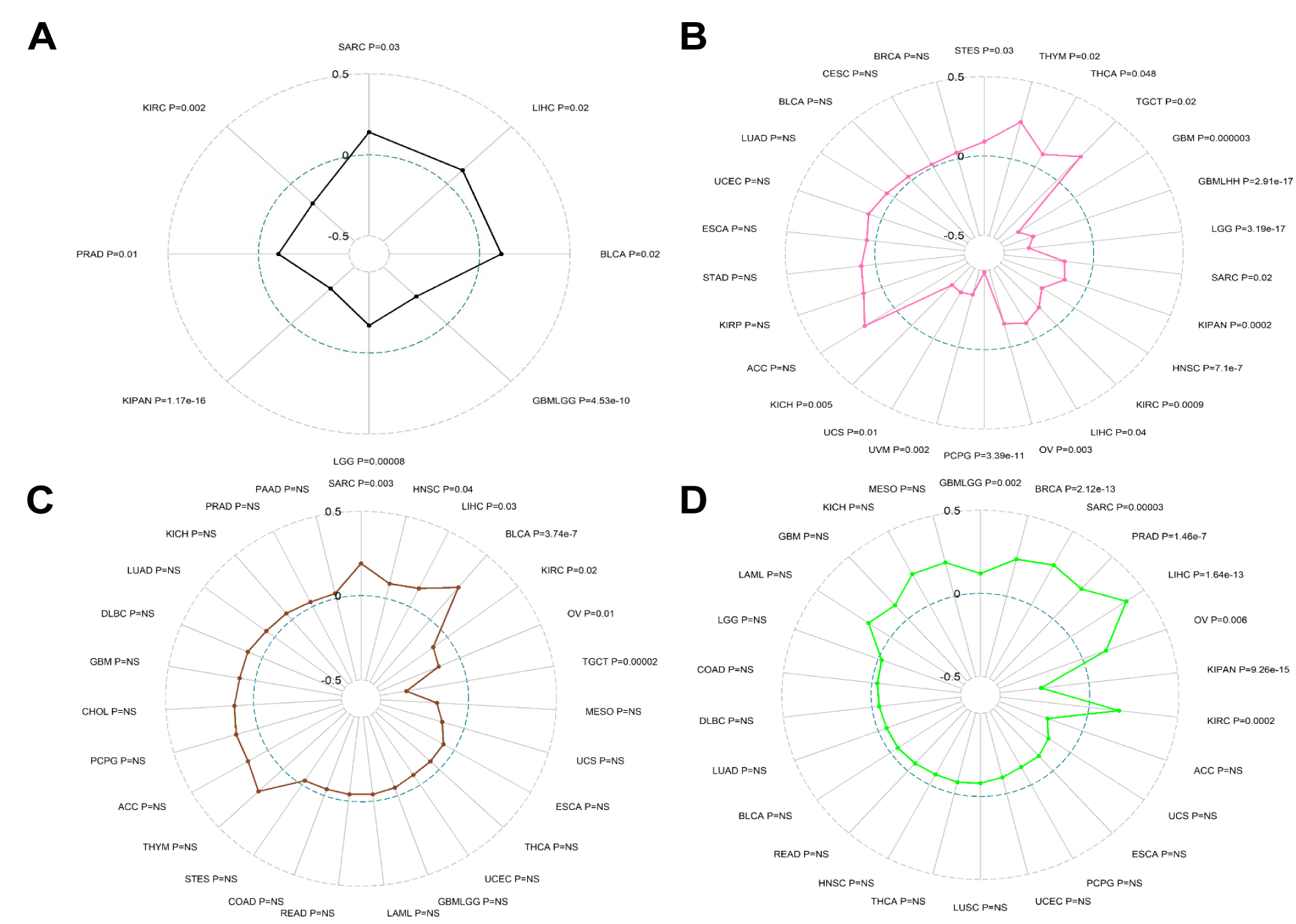

Supplement: Supplementary file 3 [file Image_3.tif]

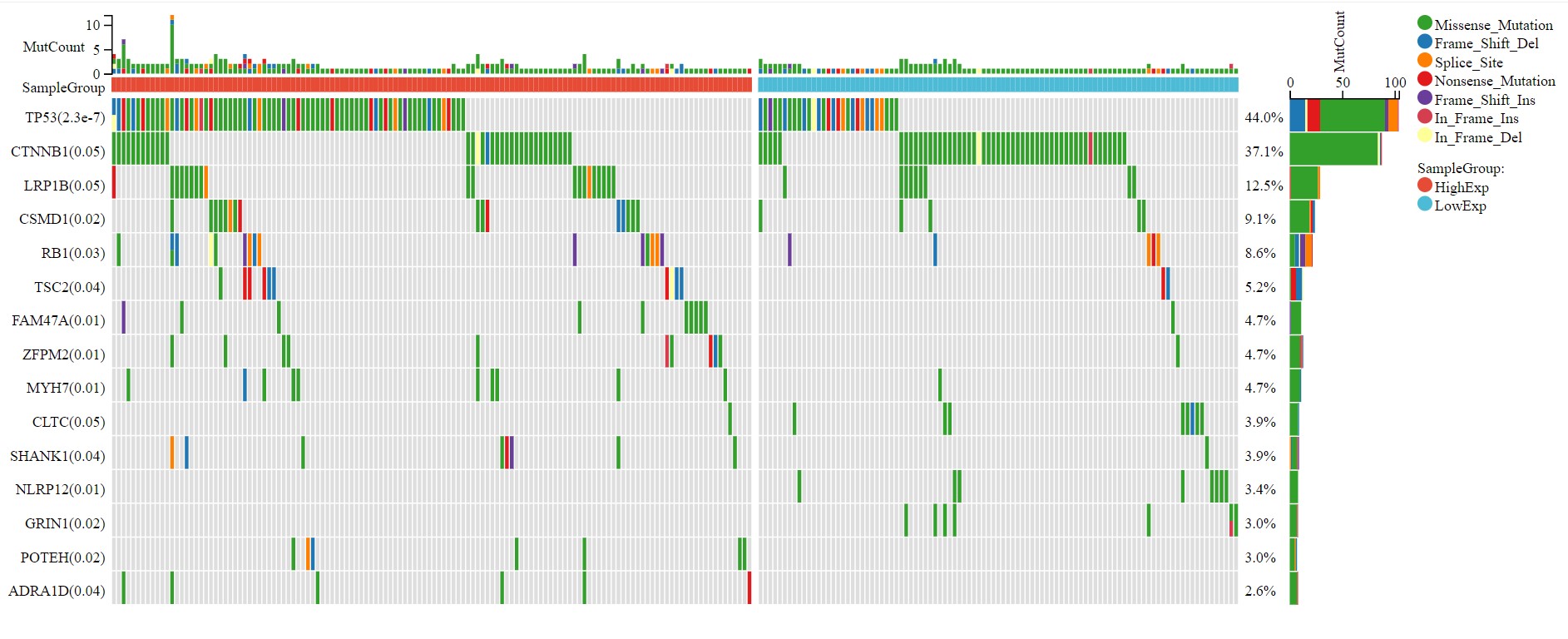

Supplement: Supplementary file 4 [file Image_4.jpeg]
